# The Alcohol Policy Information System (APIS) and Policy Research At NIAAA

**Published:** 2011

**Authors:** Gregory Bloss

Public policies have the potential to prevent the adverse consequences of alcohol consumption on a larger scale than any other category of interventions. However, measuring the effects of specific policies on alcohol-related behaviors and health outcomes is difficult and presents a variety of daunting challenges. One important challenge stems from the nonexperimental nature of most policy research, which makes it difficult to distinguish between causal relationships and noncausal associations. Another key challenge arises from the complexity of alcohol-related behaviors and outcomes and the wide range of potential effects that specific policy interventions may have on different groups and actors in various contexts. A third important challenge involves the difficulty in accurately characterizing the policies to be studied, which can be attributed largely to the arcane legal framework of statutes and regulations in which policies are created. This challenge is magnified by the enormous variety of alcohol-related public policies that have been adopted at all levels of government and the myriad variations in specific provisions that are embedded in the laws and regulations. Valid analysis of policy effects depends on surmounting all of these challenges and accurately characterizing policies and discerning the true causal effects of those policies on well-specified outcomes of interest.

The Alcohol Policy Information System (APIS) (http://alcoholpolicy.niaaa.nih.gov) was created by the National Institute on Alcohol Abuse and Alcoholism (NIAAA) as a tool to facilitate research on the effects and effectiveness of alcohol-related public policies by providing authoritative, detailed, and comparable information on alcohol-related policies at the State and Federal levels in the United States. APIS data is based on primary legal research on the statutes and regulations through which policies are established. APIS provides detailed coverage for 35 specific policy topics organized in eight categories:

## Underage Drinking

- Possession/Consumption/Internal Possession
- Purchase
- Furnishing
- Age of Server-On-Premises
- Age of Seller-Off-Premises
- Use/Lose: Driving Privileges
- Hosting Underage Drinking Parties
- False Identification

## Blood Alcohol Concentration (BAC) Limits

- Adult Drivers
- Drivers Under 21
- Recreational Boaters

## Transportation

- Open Container
- Vehicular Insurance: Losses Attributed to Intoxication

## Taxation

- Beer Taxes
- Wine Taxes
- Distilled Spirits Taxes
- Sparkling Wine Taxes
- Flavored Alcoholic Beverages Taxes

## Retail Sales

- Keg Registration
- Beverage Service Training
- Sunday Sales

## Alcohol Control Systems

- Beer (Retail)
- Beer (Wholesale)
- Wine (Retail)
- Wine (Wholesale)
- Distilled Spirits (Retail)
- Distilled Spirits (Wholesale)

## Pregnancy and Alcohol

- Warning Signs: Drinking During Pregnancy
- Criminal Prosecution
- Civil Commitment
- Priority Treatment
- Child Abuse/Neglect
- Reporting Requirements

## Health Care Services and Financing

- Health Insurance: Losses Attributed to Intoxication (“UPPL”)
- Health Insurance Parity

The coverage period for most topics begins January 1, 1998, and extends through January 1, 2010, with an update to January 1, 2011, to be posted in the coming months. For each policy topic, APIS provides detailed comparison tables showing both up-to-date policy information and policy changes over time, with exact effective dates for changes that took effect during the coverage period. APIS also provides descriptive overviews, maps and charts, summaries of relevant Federal law, legal citations, and detailed explanatory notes, as well as State profiles of the various laws that address underage drinking in each State.

APIS was developed as a tool to support research on the effects and effectiveness of alcohol-related public policies. Policy topics covered in APIS were chosen on the basis of several considerations, including public health significance, the salience of the research area, and the feasibility of legal research on State-level statutes and regulations to discern valid and meaningful policy characteristics and differences, both across jurisdictions and over time. Policies established at local (i.e., county and municipal) levels of government and policies established by case law are outside the scope of APIS. As a result, policies that vary substantially in these dimensions (for instance, restrictions on the days and hours of legal sale, which varies at the local level, and dram-shop liability, which is established in many States only through case law) are not covered within APIS.

NIAAA funds a variety of research projects on the effects of alcohol-related public policies and encourages new applications for research grants in this area. A recent set of funding opportunity announcements, titled “Research on Alcohol-Related Public Policies Such As Those Detailed in the Alcohol Policy Information System” (PA–11–087, PA–11–088, and PA–11–089, for R01, R03, and R21 applications, respectively), describes a wide range of research projects that could be supported in this area. Information on applying for these and other research grants is available at http://www.niaaa.nih.gov/ResearchInformation/ExtramuralResearch/default.htm.
